# Forecasting the spread of COVID-19 using LSTM network

**DOI:** 10.1186/s12859-021-04224-2

**Published:** 2021-06-10

**Authors:** Shiu Kumar, Ronesh Sharma, Tatsuhiko Tsunoda, Thirumananseri Kumarevel, Alok Sharma

**Affiliations:** 1 School of Electrical and Electronics Engineering, Fiji National University, Suva, Fiji; 2 Laboratory for Medical Science Mathematics, Department of Biological Sciences, Graduate School of Science, University of Tokyo, Tokyo, 113-0033 Japan; 3 Laboratory for Medical Science Mathematics, RIKEN Center for Integrative Medical Sciences, Yokohama, 230-0045 Japan; 4 Department of Medical Science Mathematics, Medical Research Institute, Tokyo Medical and Dental University (TMDU), Tokyo, 113-8510 Japan; 5 Laboratory for Transcription Structural Biology, RIKEN Center for Biosystems Dynamics Research, 1-7-22 Suehiro, Tsurumi-ku, Yokohama, Kanagawa 230-0045 Japan; 6 Institute for Integrated and Intelligent Systems, Griffith University, Nathan, Brisbane, QLD Australia

**Keywords:** COVID-19, Long short-term memory (LSTM), End date prediction, Pandemic

## Abstract

**Background:**

The novel coronavirus (COVID-19) is caused by severe acute respiratory syndrome coronavirus 2, and within a few months, it has become a global pandemic. This forced many affected countries to take stringent measures such as complete lockdown, shutting down businesses and trade, as well as travel restrictions, which has had a tremendous economic impact. Therefore, having knowledge and foresight about how a country might be able to contain the spread of COVID-19 will be of paramount importance to the government, policy makers, business partners and entrepreneurs. To help social and administrative decision making, a model that will be able to forecast when a country might be able to contain the spread of COVID-19 is needed.

**Results:**

The results obtained using our long short-term memory (LSTM) network-based model are promising as we validate our prediction model using New Zealand’s data since they have been able to contain the spread of COVID-19 and bring the daily new cases tally to zero. Our proposed forecasting model was able to correctly predict the dates within which New Zealand was able to contain the spread of COVID-19. Similarly, the proposed model has been used to forecast the dates when other countries would be able to contain the spread of COVID-19.

**Conclusion:**

The forecasted dates are only a prediction based on the existing situation. However, these forecasted dates can be used to guide actions and make informed decisions that will be practically beneficial in influencing the real future. The current forecasting trend shows that more stringent actions/restrictions need to be implemented for most of the countries as the forecasting model shows they will take over three months before they can possibly contain the spread of COVID-19.

**Supplementary Information:**

The online version contains supplementary material available at 10.1186/s12859-021-04224-2.

## Background

Coronavirus disease (COVID-19) emerged in December 2019 and within three months, it was declared a global pandemic [1]. COVID-19 represents a diverse family of positive RNA virus capable of causing severe respiratory disease in humans and animals [2]. COVID-19 is a new member of beta coronavirus and is related to severe acute respiratory syndrome coronavirus (SARS-CoV) [3]. SARS-CoV emerged in 2002 and spread to 26 countries, infecting more than 8000 patients. SARS-CoV was eventually contained through public health measures, as there was no approved vaccine for SARS-CoV infections. With the second phase of a smaller outbreak in 2004, coronavirus has since not affected humans to that extent after the initial outbreak ended. However, SARS-CoV-like virus continued to circulate in bats and to date, not much effort has been made to treat and control coronavirus infections.

A coronavirus outbreak is a threat to the global economy and health security. The outbreak is likely to continue for months as there is no treatment currently available to prevent COVID-19 infections. Compared to SARS-CoV, COVID-19 exhibits faster human-to-human transmission, leading to the declaration of a global pandemic with millions of infections and thousands of deaths worldwide. The rapid spread of COVID-19 disease and its impact on the economy has emphasized the development of coronavirus vaccines. To this end, the research on COVID-19 global pandemic and future growth is a hot topic, and many studies are being conducted on the preventive measures of COVID-19. On the other hand, the rush towards vaccine development is in progress and may take many months for its validation and test. With the current situation of lockdown, infections, and deaths, it is crucial to know when the spreading of COVID-19 will be over.

With the current number of COVID-19 cases for each country, the trend shows that the cases per day is still increasing for a number of the countries, and it is not clear by when the spread of COVID-19 can be contained. If the dates by when COVID-19 can be contained is known for each country, it will help the government, policymakers, entrepreneurs and businesses make appropriate decisions that could potentially impact public and social matters. To address the above concerns, computational techniques [4–6] can be applied to model the spread of COVID-19 and forecast the dates by when a country might be able to contain the spread of COVID-19. In [6], the authors have used the fractional-order susceptible individuals, asymptomatic infected, symptomatic infected, recovered, and deceased (SEIRD) model for forecasting the spread of COVID-19. Using Italy’s data, they showed that the fractional-order model has lower root-mean-square error (RMSE) than the classical one. The authors in [5] proposed a autoregressive integrated moving average (ARIMA) model for forecasting the expected number of daily new cases of COVID-19 in Saudi Arabia. They forecasted the COVID-19 cases for the next 4 weeks. Four different models were evaluated namely the autoregressive model, moving average, a combination of both autoregressive moving average and ARIMA. They found out that the ARIMA model performed well using Saudi Arabia data. Models from the exponential smoothing family is used for forecasting the cumulative daily confirmed cases, deaths and recoveries globally in [4]. Exponential smoothing models is used as the authors claim that it can capture a variety of trend, seasonal forecasting patterns and is mostly suitable for short time series data. An autoregressive neural network approach has been proposed by Saba and Elsheikh [7] for the prediction of the prevalence of COVID-19 outbreak in Egypt. Compared to the officially reported cases, a good performance was noted for their approach. One of the studies [8] also revealed a significant relationship between air pollution and COVID-19 infection. In [9], the authors proposed a long short-term memory (LSTM) network for forecasting the air quality in ten different horizons in Delhi, India. Several approaches [10–14] have been recently proposed using deep learning methods for forecasting the spread of COVID-19. In [11], Chimmula and Zhand proposed an LSTM model for forecasting the number of COVID-19 cases in Canada. They predicted that Canada will be able to end the COVID-19 outbreak around June 2020. Their prediction was somewhat close as the number of cases was reducing from May 2020 before undergoing a second wave of COVID-19 outbreak. The transmission rate of Canada was also compared with that of the USA and Italy. Chandra, Jain and Chauhan [10] proposed three different approaches using LSTM, bidirectional LSTM and encoder-decoder LSTM models for forecasting the spread of COVID-19 infections among numerous selected states in India. Both univariate and multivariate models were considered. For multivariate model, the authors used input from the state that is considered and inputs from three adjacent states. The authors reported that the LSTM model performed well for most of the cases in comparison to the bidirectional LSTM and encoder-decoder LSTM models. A rolling mean of 3-days has been used for processing the data. Promising results were noted, and the approach can be used for forecasting the spread of COVID-19 infections in other countries or areas.

In this study, we obtain daily new cases and apply the long short-term memory (LSTM) architecture for forecasting the dates by when a country might be able to contain the spread of this novel coronavirus. The term ‘contain the spread of COVID-19’, refers that a country will be able to bring down its daily new cases of COVID-19 to less than 1% of its RMSE. We believe that the country will be able to control and manage the spread of any new cases of COVID-19 in future (if any) after it achieves cases less than 1% of its RMSE unless there are changes in travel and other restriction measures. The results obtained are promising and shows the forecasted number of new cases in the coming months for each country. The results also suggest that countries should not haste into easing restrictions as it can result in a second wave of COVID-19 outbreak in the country.

## Results

**Fig. 1:**
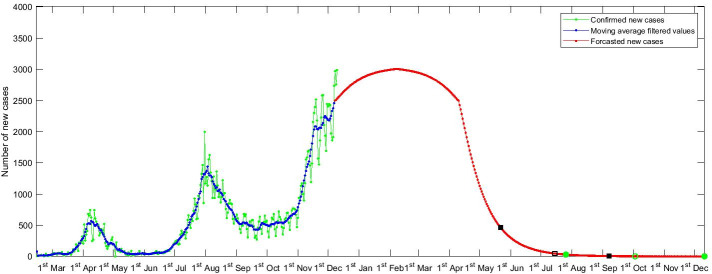
Prediction of the expected number of new cases of COVID-19 for Japan

The COVID-19 data used in this work has been obtained from the World-o-meter website [15] and we have extracted the cumulative total new cases data for each day from 15th February to 13th December, 2020. To test the reliability of our proposed model, we first used 80% of the data for training, 10% of the data for validation while the remaining 10% used for evaluating the model. The root mean squared error (RMSE) obtained for the different countries is shown in Table 1. We then used all the available data (15th February–13th December) to retrain the model and predict the expected number of cases for the near future. From our prediction model, we have extracted the range of dates when no new cases are expected, i.e. a country might be able to contain the spread of COVID-19. Figure 1 shows the predicted number of daily new cases of COVID-19 for Japan. For calculating the range of the dates by when a country might be able to contain the spread of COVID-19, we have used 1% of RMSE on each side of the date when first zero case is predicted by our proposed model to indicate the dates between which it is expected that a country will be able to contain the spread of COVID-19. The filled green circles indicate the range while the unfilled green circle indicates when the first zero case of COVID-19 might be recorded. We have used 1% of RMSE for stating the range to keep the range of the dates within a few months.

**Table 1:** The RMSE values are obtained on the test set with 80% of data used for training, 10% used for validation and the remaining 10% of the data used as the test set

	Country	RMSE	Forecasted dates by when COVID-19 might be contained
1	New Zealand	2	4th January–3rd March, 2021
2	Australia	9	16th January–23rd February, 2021
3	United States	9692	20th December, 2021–11th May, 2022
4	France	17,121	12th March–4th June, 2021
5	Germany	12,837	19th February–30th August, 2021
6	Italy	13,193	4th February–29th April, 2021
7	Russia	8090	22nd March–10th October, 2022
8	Spain	1993	22nd March–30th June, 2021
9	United Kingdom	5944	26th March–23rd August, 2021
10	Mexico	2197	5th April–10th July, 2021
11	Japan	933	26th July–11th December, 2021
12	India	16,063	29th March–17th July, 2021
13	Brazil	7069	17th May–11th August, 2021
14	Turkey	11,617	1st April–3rd August, 2021
15	Iran	5110	26th January–2nd May, 2021

Prediction of the dates is made by using all the available data for training the model

## Discussion

To validate and show the reliability of our proposed model we have used the data for New Zealand since New Zealand has already been able to contain the spread of COVID-19 and has been able to achieve zero new cases of COVID-19. Therefore, for New Zealand, we have used the data from 15th February to 16th April (61 days of data) to predict the dates between which the number of new cases of COVID-19 will fall below 5% of RMSE and when the number of new cases of COVID-19 will become less than 1% of its RMSE. Our proposed model forecasted that the number of new cases of COVID-19 (for New Zealand) would become less than 1% of its RMSE between 25th April and 15th May. Comparing this with the facts, New Zealand recorded its first zero daily new cases of COVID-19 on 13th May, although a few new cases were reported days later. This shows that our model was able to give a reliable prediction of the dates when New Zealand’s new cases of COVID-19 would fall below 1% of its RMSE. However, since there were ease of travel and other restriction measures in New Zealand it suffered a 2nd wave of COVID-19 cases. Therefore, to predict when it will again be able to contain the spread of COVID-19, we have used all the data from 15th February to 13th December, 2020 to forecast when it will be able to contain the 2nd wave of COVID-19. In [16], a susceptible-infected-recovered (SIR) model has been used for the prediction of the end date of COVID-19. And for New Zealand, their model predicted (using data till 25th April) that it will be able to contain the spread of COVID-19 by 10th May. However, New Zealand was able to contain the spread of COVID-19 by 21st May (although a 2nd outbreak occurred later in August), which was more closely predicted by our proposed model. For Australia and Japan, the dates predicted by [16] were 23rd May and 26th September, respectively. On the other hand, our proposed model predicted (using data till 25th April to make a fair comparison) that Australia and Japan might be able to contain the spread of COVID-19 between 1st June–5th July and 24th May–2nd July, respectively. Given the figures, Australia was not able to contain the spread of COVID-19 by 23rd May as predicted by [16], neither was it able to contain the spread of COVID-19 by 5th July as predicted by our model. Nevertheless, it should be noted that Australia was able to control and reduce new cases of COVID-19 to less than 5 between 6 and 9th June, however, ease of restrictions resulted in the second wave of COVID-19. Therefore, it can be said that our proposed model is more reliable because if restrictions were not lifted, Australia might have been able to contain the spread of COVID-19 by 5th July. This also suggests and shows that lifting restrictions in the early stages can result in a second wave of COVID-19 outbreak. This is the trend seen by Japan (as shown in Fig. 1) and quite a number of other countries as shown in the Additional file 1: Figures S1–S14. Furthermore, promising results were also obtained by [10]. However, we cannot directly compare all the results as the authors looked at forecasting the spread of COVID-19 infections for numerous states in India, whereas our work considered forecasting the spread of COVID-19 infections for numerous countries. They also forecasted the spread of COVID-19 infections for the whole of India. Their proposed LSTM model forecasted a mean of around 30,000–35,000 daily new cases by end of January 2021 for India with uncertainty being in the range of about 22,500–40,000. On the other hand, our model forecasted that India’s daily new cases will drop to around 3000 with uncertainty being in the range of 0 to about 16,000. Considering the fact that at the end of January 2021 the actual daily new cases of COVID-19 was around 12,000, we can say that our proposed model is more reliable and accurate in forecasting the spread of COVID-19 infections.

Looking at the forecasted dates of when the countries might be able to contain the spread of COVID-19, it can be said that some of the countries may need to take more stringent actions to be able to contain COVID-19 or become COVID-19 free. It is also recommended that countries should not rush into easing restriction measures as it can lead to another outbreak in the country. The prediction results of when a country will be able to bring down its daily new cases to less than 5% of the RMSE value is given in Additional file 1: Table S1. Furthermore, the prediction results will be updated monthly on our website: http://www.alok-ai-lab.com/materials.php to reflect any change in restriction measures placed by the countries as it will have a direct impact on the containment of COVID-19.

## Conclusions

Computational models are entirely data-oriented with the predictions being made based on the past data. Therefore, our model's prediction is based on the current situation, and if new restrictions or measures are implemented, then the model will need to be retrained. The results of our model can be used by policymakers, business partners and entrepreneurs to make informed decisions with some reliable knowledge about future cases of COVID-19.

## Methods

The knowledge about the possible number of new cases of COVID-19 in the future will be of great importance as it will provide an insight about steps that can be taken considering the disease and its implications and will help in making informed decisions. To this end, we propose the LSTM network-based model for the prediction of the expected number of new cases of COVID-19 in the near future.

**Fig. 2:**
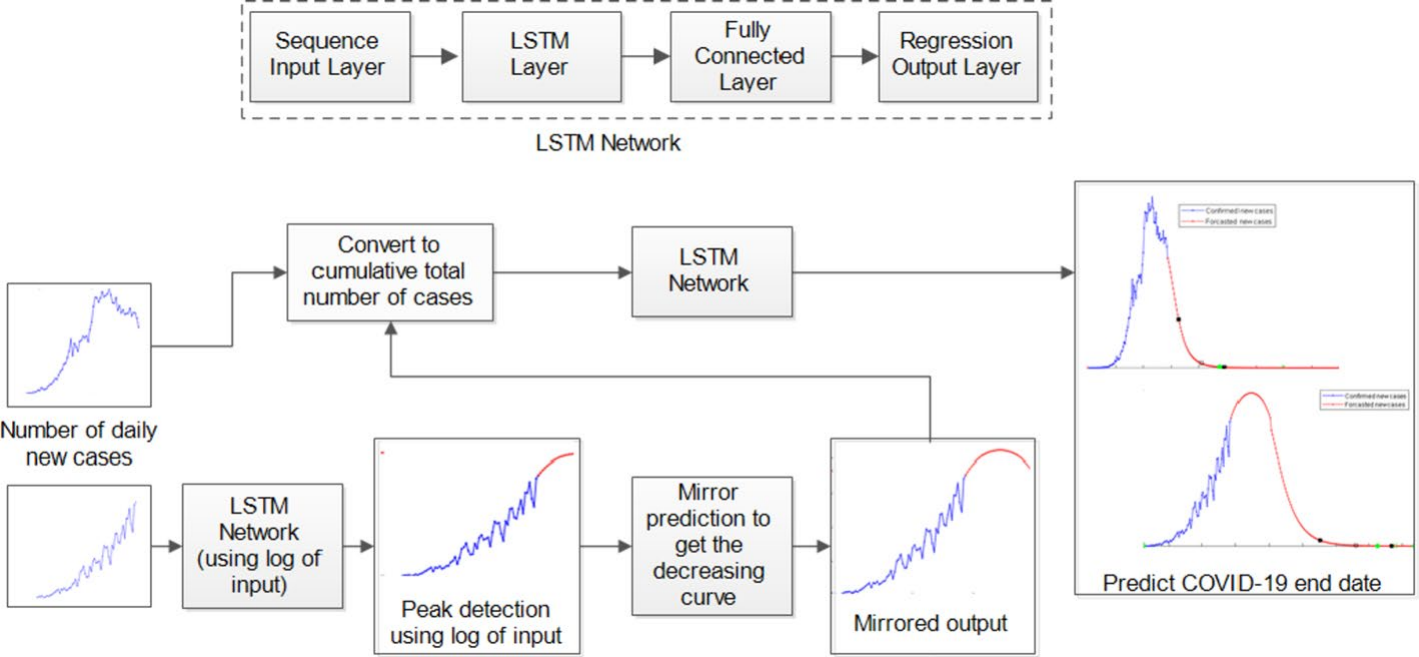
Architecture of the proposed LSTM network used in this work (on top) and the strategy of the proposed work (bottom)

Deep learning has gained widespread attention in various fields outperforming traditional methods [17–19]. LSTM network is a deep learning approach that is a recurrent neural network having an LSTM layer. LSTM uses its memory cell, which consists of the input gate, forget gate and the output gate to learn a better model making use of the information from the past. The LSTM network architecture used in this work is given in Fig. 2. It consists of a sequence input layer, followed by one LSTM layer with 150 hidden units (determined through experimentation), a fully connected layer and a regression layer at the output. The sequence input layer is used to provide the time series input to the network. The LSTM layer learns long-term dependencies between a series of time series data to predict the future value at the next time-step. In this work, we have adopted 2 different strategies to predict when a country might be able to contain the spread of COVID-19 depending on whether their number of daily new cases of COVID-19 are increasing or decreasing as shown in Fig. 2 (bottom). For countries, whose number of daily new cases of COVID-19 are decreasing, we have used the cumulative number of confirmed cases per day as the time series input to the LSTM network. The time-step used is one day and the model predicts the cumulative number of cases for the next day. Our model then uses the predicted value of the cases for the next day, updates its states and then uses this predicted value to estimate the expected number of cases for the following day. This procedure is repeated for predicting the number of new cases that are expected in the coming months until a country might be able to contain the spread of COVID-19. For countries whose number of daily new cases of COVID-19 are increasing, we performed a 2-phase procedure. In the first phase, the log of the number of daily new cases of COVID-19 is used to determine the peak point (pre-processing is performed using 7-point moving average filter). In this phase, we obtain the predicted number of daily new cases of COVID-19 until the expected peak point is reached. The second phase is same as the procedure used for the countries whose number of daily new cases of COVID-19 are decreasing, however, the predicted values of daily new cases of COVID-19 obtained in phase 1 are used as the observed points. We have used the cumulative number of confirmed new cases per day for countries whose number of daily new cases of COVID-19 are decreasing as the model can easily learn the decreasing trend using this data. On the other hand, we have 1st used the log of the number of daily new cases of COVID-19 to determine the peak point for countries whose number of daily new cases of COVID-19 are increasing. This is because the graph of log approaches an asymptote, which makes it easier to determine the peak point, while daily new cases of COVID-19 is used as it gave more accurate results compared to using cumulative number of confirmed new cases per day. Furthermore, we have carried out several experiments to determine the best moving average filter to be used. We have varied the size of the moving average filter from 3 to 21-point moving average filter, including experiment without using moving average filter and  7-point moving average filter produced the best results (lowest RMSE). Therefore, we have employed a 7-point moving average filter in our work. This accounts for the delay effects in diagnosing COVID-19 cases and any inaccuracies while curating that data.

**Fig. 3:**
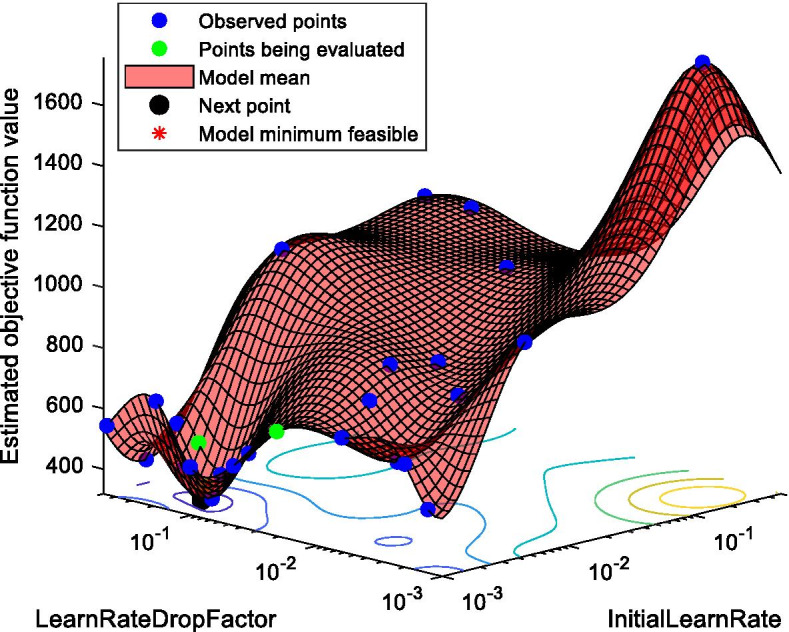
Determining the best feasible hyper-parameters of LSTM network using Bayesian optimization

Moreover, to reduce overfitting and improve the generalization of our proposed model, we have employed Bayesian optimization to tune the hyper-parameters (initial learn rate and learn rate drop factor) of the LSTM network. The range of the initial learn rate and learn rate drop factor were set to [1@e-3 5@e-1] and [1@e-3 5@e-1], respectively. These parameters were set around the default parameters. The 1st 80% of the data was used for training and the next 10% data was used for validating the performance of the LSTM network while tuning the hyper-parameters. The last 10% of the data was used as test data and used to calculate the RMSE values. Figure 3 shows the effect of selecting different values for the initial learn rate and learn rate drop factor hyper-parameters using Japan’s data. It shows how Bayesian optimization technique can find the best feasible values for these two hyper-parameters and justifies the need for optimizing the network parameters. The parameters learnt using Bayesian optimization are given in Additional file 1: Table S2.

## Supplementary Information

**Additional File 1**. Supplementary Material for forecasting the spread of COVID-19 using LSTM network.

## Data Availability

The dataset used in this study is publicly available at https://www.worldometers.info/coronavirus/

## Abbreviations

LSTM: Long short-term memory
SARS-CoV: Severe acute respiratory syndrome coronavirus
RMSE: Root mean squared error

## References

[1] Cucinotta D, Vanelli M (2020). WHO declares COVID-19 a pandemic. Acta Biomed.

[2] Woo PCY, Huang Y, Lau SKP, Yuen K-Y (2010). Coronavirus genomics and bioinformatics analysis. Viruses.

[3] Mohammadi M, Meskini M, do Nascimento Pinto AL (2020). 2019 Novel coronavirus (COVID-19) overview. J Public Health.

[4] Petropoulos F, Makridakis S (2020). Forecasting the novel coronavirus COVID-19. PLoS ONE.

[5] Alzahrani SI, Aljamaan IA, Al-Fakih EA (2020). Forecasting the spread of the COVID-19 pandemic in Saudi Arabia using ARIMA prediction model under current public health interventions. J Infect Public Health.

[6] Rajagopal K, Hasanzadeh N, Parastesh F, Hamarash II, Jafari S, Hussain I (2020). A fractional-order model for the novel coronavirus (COVID-19) outbreak. Nonlinear Dyn.

[7] Saba AI, Elsheikh AH (2020). Forecasting the prevalence of COVID-19 outbreak in Egypt using nonlinear autoregressive artificial neural networks. Process Saf Environ Prot Trans Inst Chem Eng B.

[8] Zhu Y, Xie J, Huang F, Cao L (2020). Association between short-term exposure to air pollution and COVID-19 infection: evidence from China. Sci Total Environ.

[9] Tiwari A, Gupta R, Chandra R: Delhi air quality prediction using LSTM deep learning models with a focus on COVID-19 lockdown. 2021.

[10] Chandra R, Jain A, Chauhan DS: Deep learning via LSTM models for COVID-19 infection forecasting in India. 2021. 10.1371/journal.pone.0262708 PMC8797257 35089976

[11] Chimmula VKR, Zhang L (2020). Time series forecasting of COVID-19 transmission in Canada using LSTM networks. Chaos Solitons Fractals.

[12] Yang Z, Zeng Z, Wang K, Wong S-S, Liang W, Zanin M, Liu P, Cao X, Gao Z, Mai Z (2020). Modified SEIR and AI prediction of the epidemics trend of COVID-19 in China under public health interventions. J Thorac Dis.

[13] Farooq J, Bazaz MA (2021). A deep learning algorithm for modeling and forecasting of COVID-19 in five worst affected states of India. Alex Eng J.

[14] Tomar A, Gupta N (2020). Prediction for the spread of COVID-19 in India and effectiveness of preventive measures. Sci Total Environ.

[15] COVID-19 CORONAVIRUS PANDEMIC. https://www.worldometers.info/coronavirus/. Accessed 15 Dec 2020.

[16] Luo J: When will COVID-19 end? Data-driven prediction. 2020.

[17] Gao Y, Gao B, Chen Q, Liu J, Zhang Y (2020). Deep convolutional neural network-based epileptic electroencephalogram (EEG) signal classification. Front Neurol.

[18] Kumar S, Sharma A, Tsunoda T (2019). Brain wave classification using long short-term memory network based OPTICAL predictor. Sci Rep.

[19] Chambon S, Galtier MN, Arnal PJ, Wainrib G, Gramfort A (2018). A deep learning architecture for temporal sleep stage classification using multivariate and multimodal time series. IEEE Trans Neural Syst Rehabil Eng.

