# Supplementary material for: Forecasting the spread of COVID-19 using LSTM network
Source: BMC Bioinformatics. 2021 Jun 10;22(Suppl 6):316. doi: 10.1186/s12859-021-04224-2 (PMC8190741; doi:10.1186/s12859-021-04224-2)
Supplement: Supplementary file 1 — Additional File 1. Supplementary Material for forecasting the spread of COVID-19 using LSTM network. [file 12859_2021_4224_MOESM1_ESM.pdf]

Supplementary Material for Forecasting the spread of COVID-19 using LSTM Network

Here, we present the forecast (dates and figures) of when a country might be able to contain the spread of COVID-19 (for all the countries mentioned in the paper). We have also shown the range of dates between which the daily cases are expected to fall below 5% of the RMSE value. The two filled black square marks indicate the start and end points of the range of dates between which it is expected that the number of new cases of COVID-19 will fall below 5% of RMSE. The unfilled black square mark indicates when exactly (according to our model's prediction) the predicted number of new cases of COVID-19 is expected to become 5% of RMSE.

A detailed step by step procedure for the strategy used in forecasting the number of daily new cases of COVID-19 for countries whose number of daily new cases are still increasing is as follows.

Phase 1

1. Input data is the number of daily new cases of COVID-19, however, pre-processing using a 7-point moving average filter is performed.
2. The filtered data are then used to train the LSTM network.
3. The trained LSTM network is used to predict the expected number of new cases of COVID-19 for the next day.
4. The network states are updated using the predicted value obtained in step 3, and then this predicted value is used as an observed point to determine the expected number of new cases of COVID-19 for the next day.
5. Repeat step 4 until the expected number of new cases of COVID-19 becomes steady, i.e. reaches its peak. This is determined by noting when the increase in the number of daily new cases of COVID-19 becomes less than 10.
6. The predicted values obtained to determine the peak are then mirrored and appended to the predicted values. This data is then used in the next phase

Phase 2

7. The data obtained in step 6 of phase 1 are appended to the observed daily new cases of COVID-19.
8. The running cumulative number of cases of COVID-19 is calculated (after pre-processing the data using 7-point moving average filter) and used as input to train the LSTM network.
9. Step 3 and 4 are repeated, however, the total number of cases for the next day is obtained as the predicted value. This is repeated until the peak is reached, i.e. there is no change in the total number of predicted cases.
10. The daily new cases of COVID-19 are then obtained by subtracting the next day’s cases from the previous day’s case. This data is then used to predict the range of dates (using 1% of RMSE as explained in the paper) between which it is expected that the country will be able to contain the spread of COVID-19.

Table S1: The RMSE values are obtained on the test set with 85% of data used as the training set and the remaining 15% of the data used as the test set. Prediction of the dates is made by using all the available data for training the model. Data till 13th December, 2020.

	Country	RMSE	New cases of COVID-19 will be below 5% of RMSE	Forecasted dates by when COVID-19 might be contained
1	New Zealand	2	12th December, 2020 – 5th January, 2021	4th January – 3rd March, 2021
2	Australia	9	21st December 2020 – 13th February, 2021	16th January – 23rd February, 2021
3	United States	9692	11th October, 2021 – 2nd February, 2022	20th December, 2021 – 11th May, 2022
4	France	17121	16th February – 24th March, 2021	12th March – 4th June, 2021
5	Germany	12837	6th January – 1st March, 2021	19th February – 30th August, 2021
6	Italy	13193	17th December, 2020 – 7th February, 2021	4th February – 29th April, 2021
7	Russia	8090	16th December, 2021 – 22nd May, 2022	22nd March – 10th October, 2022
8	Spain	1993	11th January – 30th March, 2021	22nd March – 30th June, 2021
9	United Kingdom	5944	2nd February – 27th April, 2021	26th March – 23rd August, 2021
10	Mexico	2197	21st February – 2nd May, 2021	5th April – 10th July, 2021
11	Japan	933	22nd May – 7th September, 2021	26th July – 11th December, 2021
12	India	16063	4th January – 10th April, 2021	29th March – 17th July, 2021
13	Brazil	7069	4th March – 31st May, 2021	17th May – 11th August, 2021
14	Turkey	11617	2nd January – 7th April	1st April – 3rd August, 2021
15	Iran	5110	19th December, 2020 – 3rd February, 2021	26th January – 2nd May, 2021

Table S2: The parameters learnt using Bayesian optimization

	Country	Initial Learn Rate	Learn Rate Drop Factor
1	New Zealand	0.0052348	0.03724
2	Australia	0.0085936	0.056028
3	United States	0.0053895<br>0.0015361	0.24922<br>0.19875
4	France	0.001017<br>0.0010011	0.056415<br>0.44165
5	Germany	0.100100<br>0.0024681	0.023417<br>0.0017341
6	Italy	0.0018091	0.39361
7	Russia	0.0045875<br>0.001007	0.019128<br>0.086192
8	Spain	0.0077356	0.050059
9	United Kingdom	0.0010163<br>0.0032498	0.010357<br>0.068014
10	Mexico	0.0019815<br>0.0021525	0.0096154<br>0.035138
11	Japan	0.018017<br>0.0023775	0.0033209<br>0.49973

12	India	0.0056832	0.26503
13	Brazil	0.0063745	0.10839
14	Turkey	0.001214	0.48957
15	Iran	0.011685	0.074179

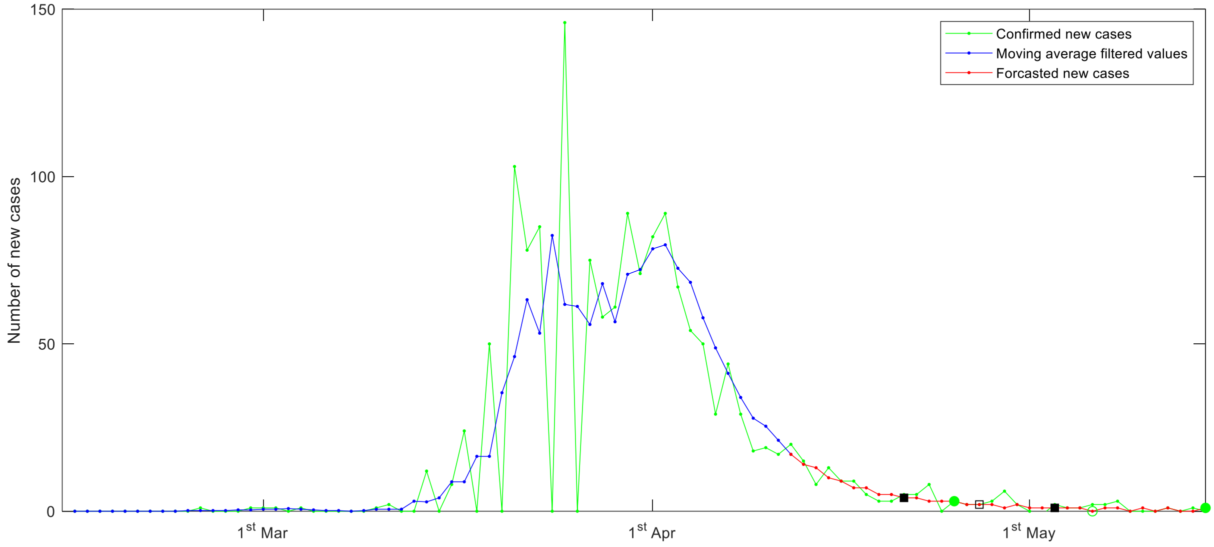

Fig. S1.1: COVID-19 forecast for New Zealand using data from 15th February to 16th April, 2020

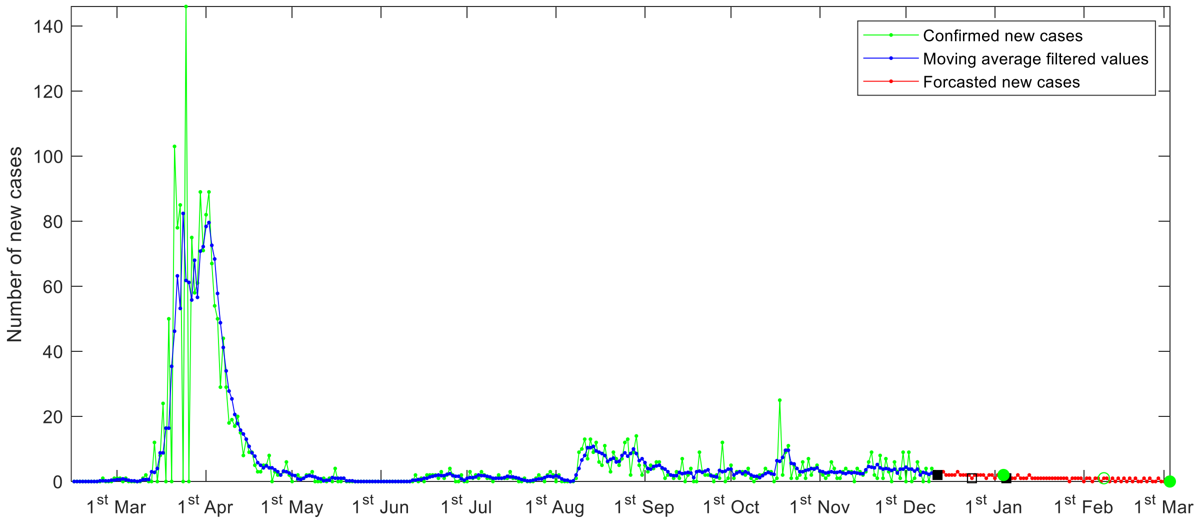

Fig. S1.2: COVID-19 forecast for New Zealand using data from 15th February to 13th December, 2020

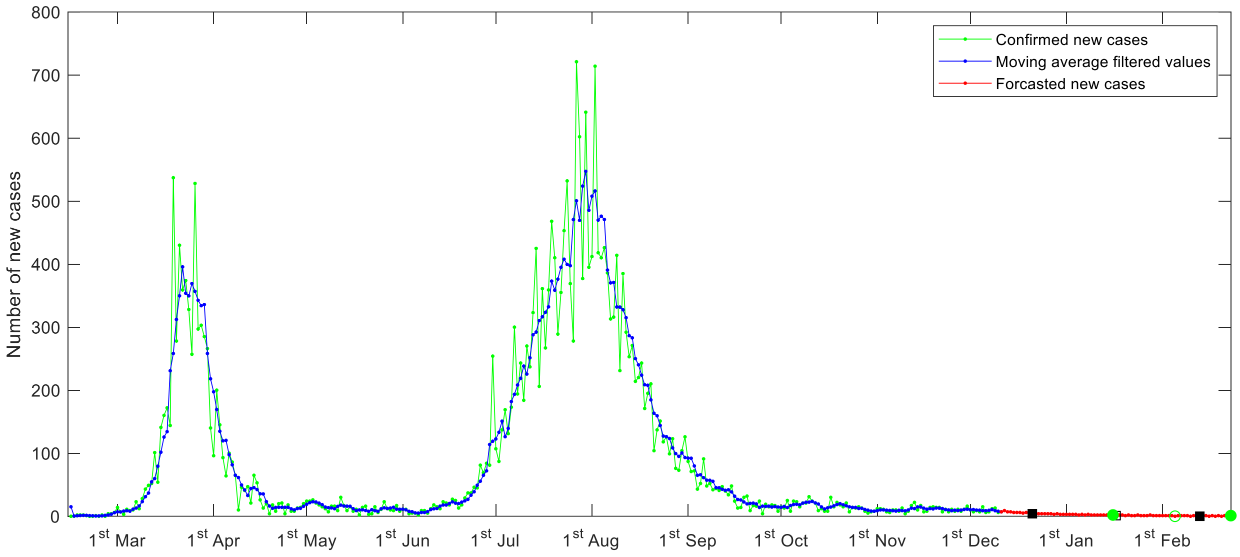

Fig. S2: COVID-19 forecast for Australia

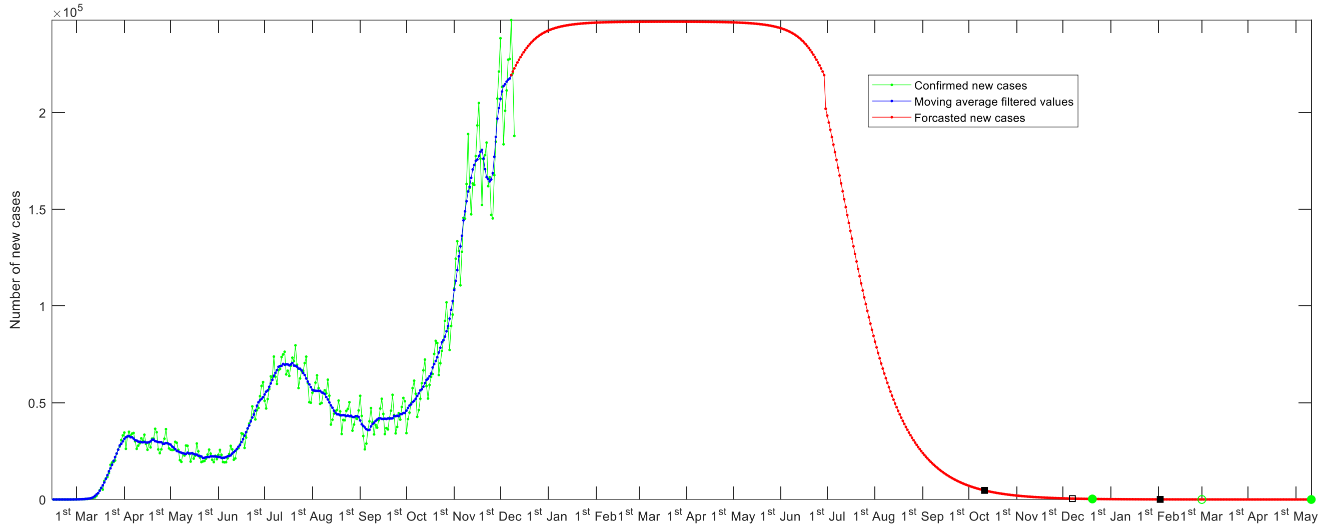

Fig. S3: COVID-19 forecast for the United States

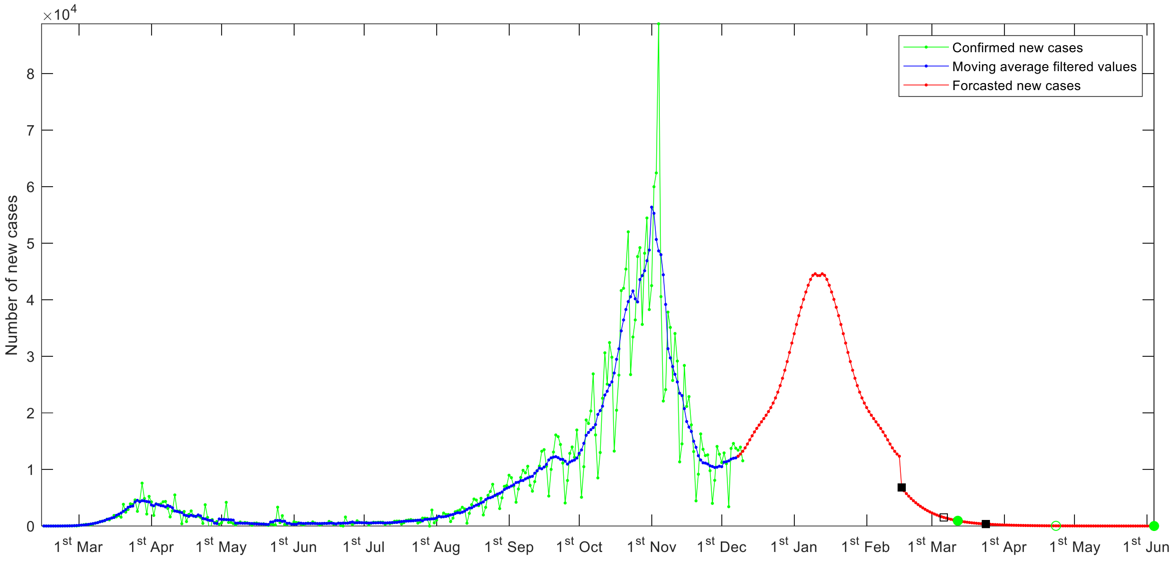

Fig. S4: COVID-19 forecast for France

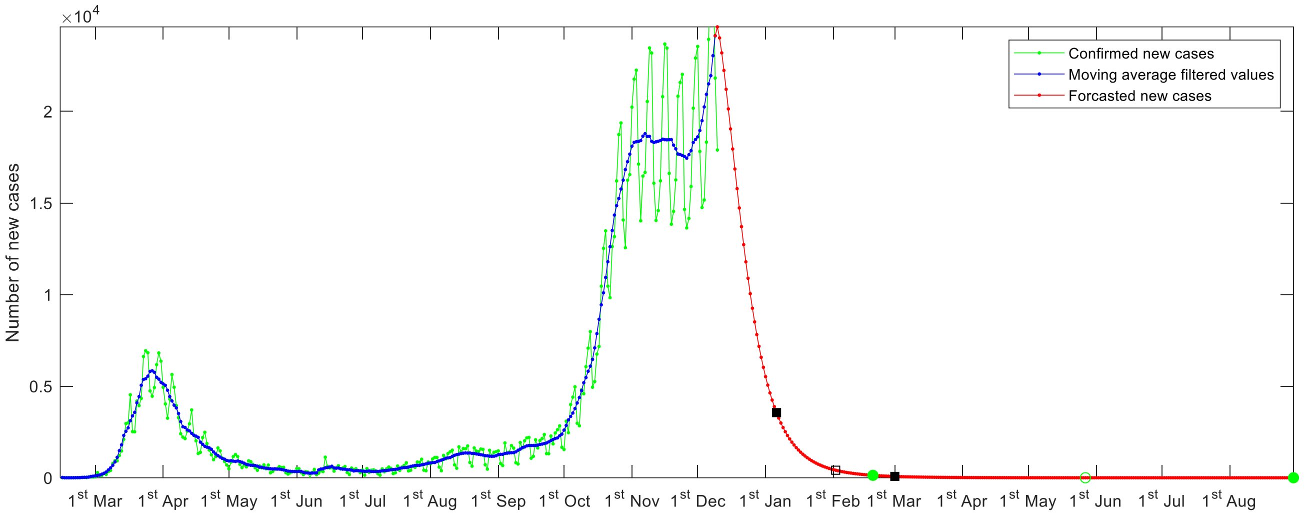

Fig. S5: COVID-19 forecast for Germany

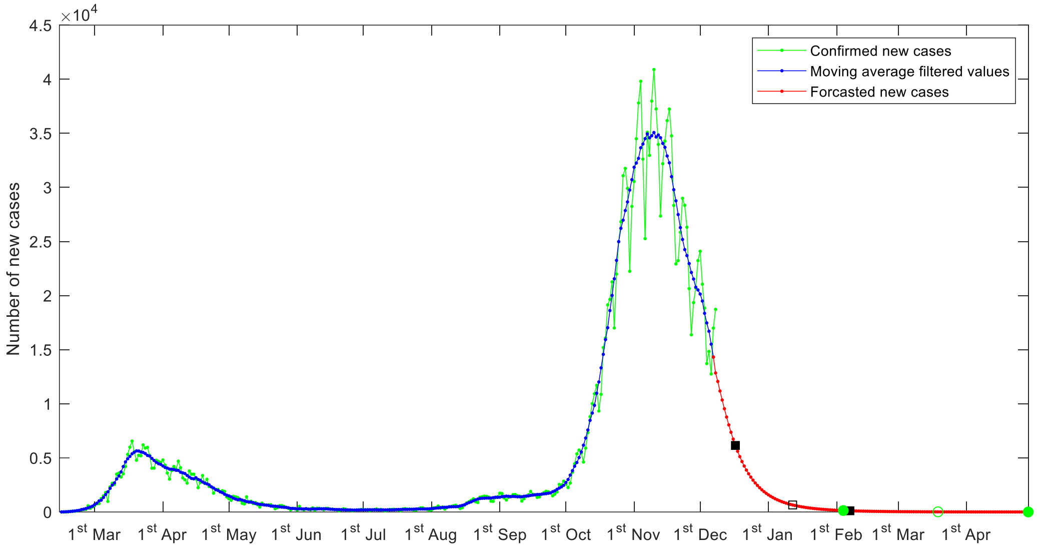

Fig. S6: COVID-19 forecast for Italy

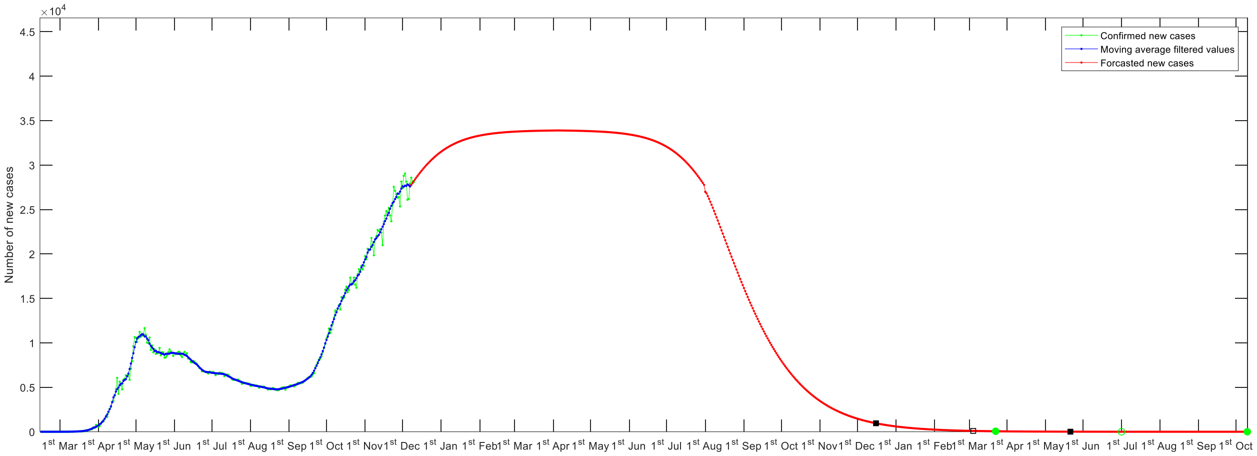

Fig. S7: COVID-19 forecast for Russia

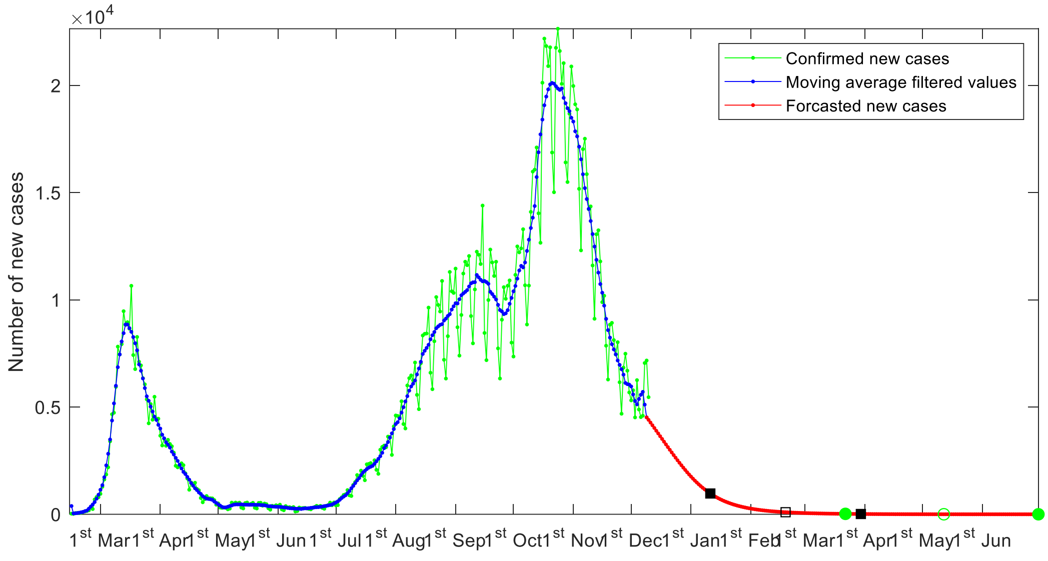

Fig. S8: COVID-19 forecast for Spain

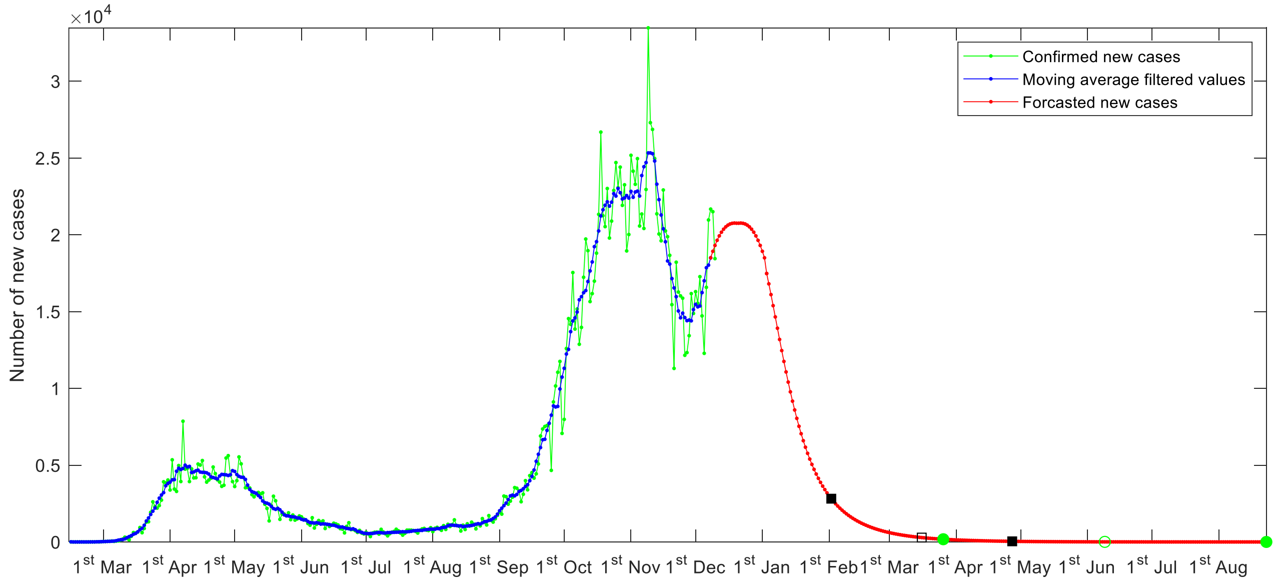

Fig. S9: COVID-19 forecast for the United Kingdom

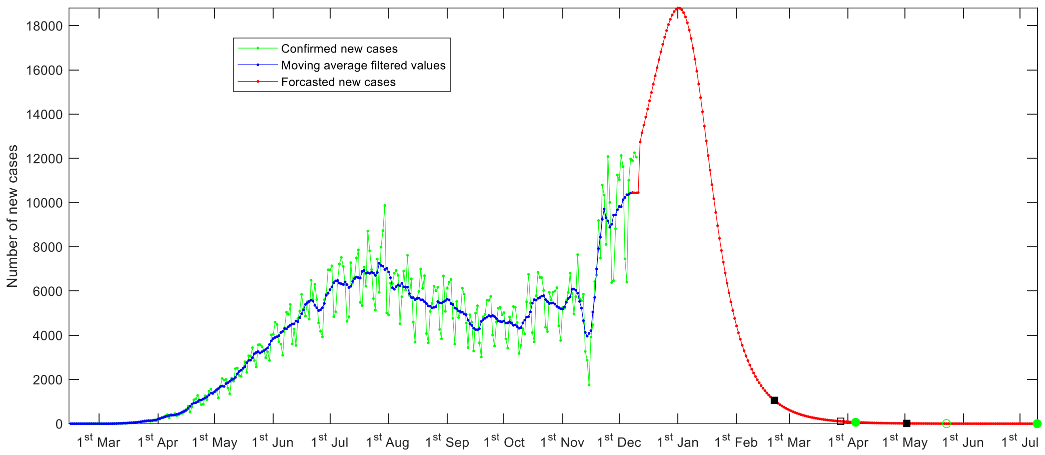

Fig. S10: COVID-19 forecast for Mexico

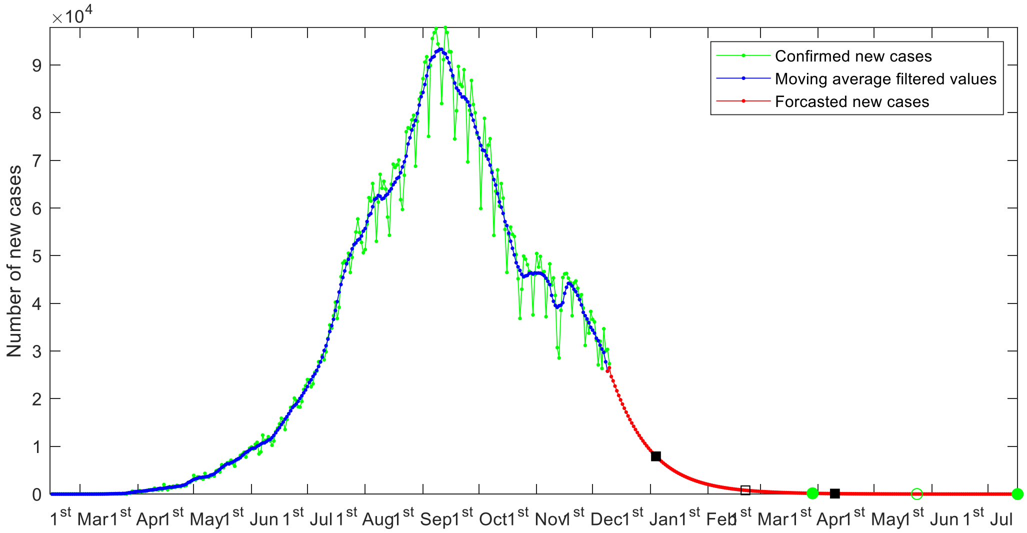

Fig. S11: COVID-19 forecast for India

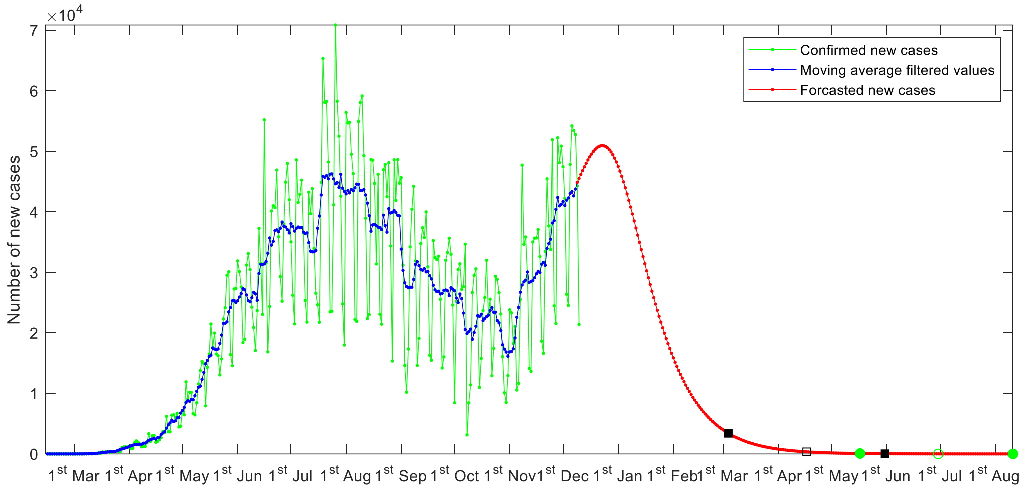

Fig. S12: COVID-19 forecast for Brazil

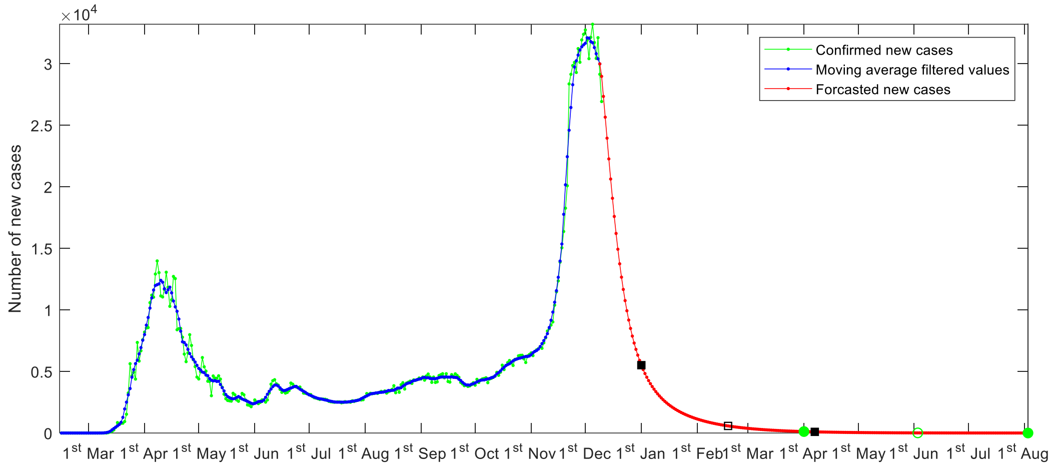

Fig. S13: COVID-19 forecast for Turkey

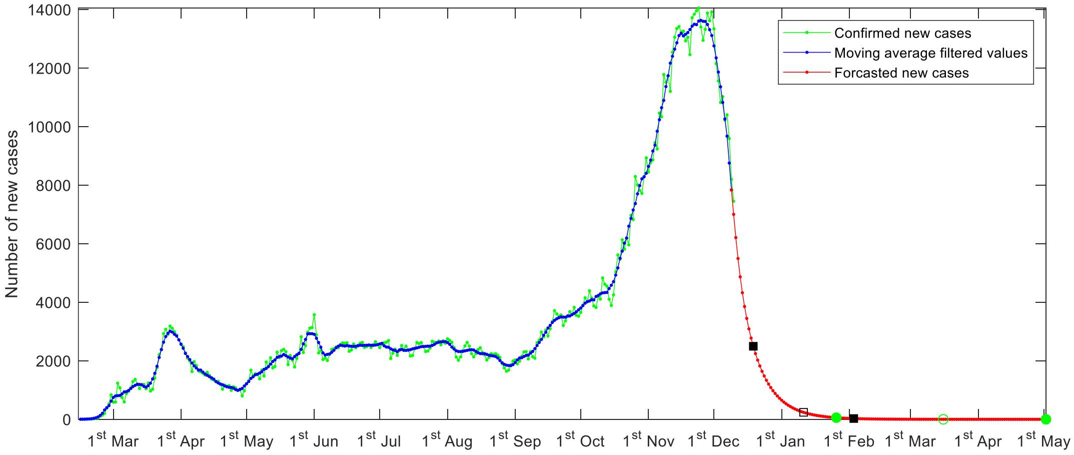

Fig. S14: COVID-19 forecast for Iran

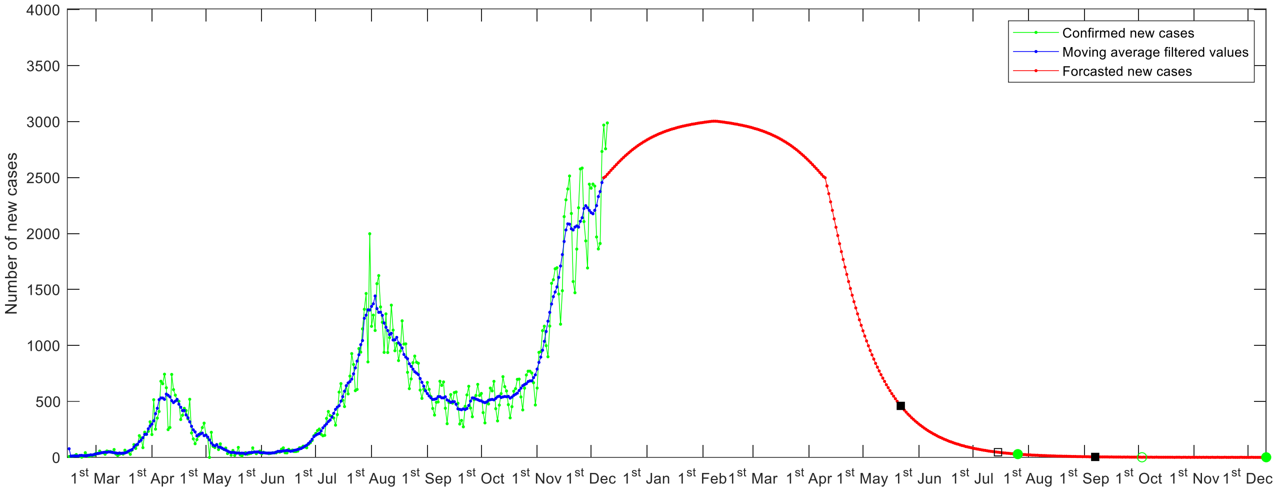

Japan
